# Professional Elevation

**Published:** 1864-03

**Authors:** A. S. Talbert

**Affiliations:** Lexington, Ky.


### DENTAL REGISTER OF THE WEST.

VOL. XVIII.] MARCH, 1864. [No. 3.

### Original Essays and Communications.

### PROFESSIONAL ELEVATION.

Read before the Mississippi Valley Dental Association, February, 1864.

BY A. S. TALBERT, D. D. S, LEXINGTON, KY.

GENTLEMEN OF THE ASSOCIATION:—With your kind indulgence, I offer a few thoughts on the status of our profession, suggesting one or two of the remedies necessary to its elevation. We, as a class, do not stand at the head of society. It is humiliating to make the confession, but while it exists we had as well acknowledge it, and set ourselves to work in earnest to remove the causes of it.

They are numerous but entirely within our control, and may be greatly remedied, if not wholly removed, by individual effort. But though numerous, the collateral, they may be reduced to two radical causes, absence of thorough education and want of correct morals. The indiscriminate practice of admitting to studentship in our offices, young men of limited education, limited means, and still more limited morals, lies at the foundation of this evil, and hinders us from taking a place on the same platform with other learned professions! It is true our Colleges and Dental Associations have done much to elevate our profession, but neither begin at the foundation, for their instructions and influences are not addressed to the primary education, nor yet to the morals of their students and members. The doors of the College are opened necessarially to all who will pay their fees, and who conduct themselves with reasonable propriety for the time being; and though a good moral character is one of the requirements to eligibility to graduation, yet how few of our Colleges would refuse to confer a degree upon one *simply on account of delinquent morals!*

It is so with our Dental Associations, formed for the promotion of dental literature and for the elevation of the standard of our profession, they have done incalculable service in the general diffusion of dental knowledge, and have had a genial influence over all their members, yet they fail to make the charlatan a man of science, nor do they discriminate between the moral and the immoral.

There should be a fitness of the means to the end, in all things You would not build an elegant superstructure on an indifferent foundation. If you would have a select party, your cards of invitation designate the character of your guests, and your doors are virtually closed against all others. So too, we must close the doors of our profession against the young man who has not attained to the requisite qualifications to fit him for a successful, honorable, and useful career. The fault is not with the colleges and associations, but with our individual selves.

The fee required of and willingly paid by the student, or the value of his services, or per chance the *show of having a student*, are too often the inducements to take them regardless of their moral qualifications or their fitness to the position to which they aspire. We should be the censors as we are the janitors of our profession.

Our experience in life qualifies us to judge of the requirements needed to success. We should advise a different avocation, when called upon by young men evidently wanting in the elements of success in the composition of their characters. I have for many years pursued this course. The consequence is, *I have no students!* no students to contribute to the success of our Dental College, of which I am proud to be numbered among the alumni. It is not that I love the College and its success the less, but that I love the honor of my profession the more.

Let every one adopt this high standard and our Colleges will teem with young men who shall be an ornament to the profession and to society. We will no longer be asked "why is it that when a man fails at everything else, and is good for nothing else, he takes to Dentistry! And who of you that has not been asked this question? Again, who of you has not been congratulated by the physician as he comes into your warm, neat, and comfortable office, upon the ease and comfort of your life as compared with his own, your freedom from exposure, your rest from your less responsible labors after office hours, while he is always liable to be called out at all hours of both day and night—the agreeable society of *your* patient while *his* is always suffering, prostration, pain, and sickness,—and yet how many physicians apply to you to take and teach their sons your profession? I have never *known one*, though it is no unusual thing for them to study medicine! Why? It is more respectable, more *honorable!* This is the accepted opinion. It is not correct, or at least should not be so.

The educated Dentist is not and should not be inferior to the physician, for to all the acquirements necessary to the successful practice of medicine, he has the *additional* qualification of the Dentist. We should, then, take a position one step higher, rather than one lower, though we may be content to place ourselves upon a level with the medical profession.

I know of no better way to attain to this than to each begin at home. See to it, that we take no students, that have not the necessary moral and intellectual qualification. It will give us caste in society, it will give us a better class of students.

The next generation of Dentists will not be such *unmit-*

*igated liars*, and quacks, as now, flourish under the assumed title of *doctor*. We will then not have so many self-made men, neither made by God or angel, but who have *sprung up* like mushrooms from the *debris* of the race, men whose impudence does more for themselves than their services do for their patient! The courtesies due to each other as members of the same calling will then be extended in confidence, and while its individual members take a higher position in society, ours will assume its proper rank among the learned professions. But I would not ignore the self-made man in the true sense of the term. I have known many examples of illustrious men, who shine in society as stars in the firmament and who are self-made men. But these all are men of moral worth, men of exalted social qualities aside from their professional attainments. To such men a higher mead of praise is due for having been *self-made*. Their severe discipline in the self taught school of experience has consumed the dross, leaving the pure metal to sparkle and shine from having been brought in contact with the asperities of real life. They are the lights of the world—the pillars of our profession—men whom we delight to honor.

Nor is moral training alone necessary. The head and the hands are to be trained as well as the heart—mind to comprehend and devise, while cunning fingers execute. The students general capability must not be overlooked, and if it is believed he has not the capacity requisite to success, he should not be encouraged to attempt it. A man may be qualified for one thing and wholly unfitted for another. A man may be capable of digging a well and not qualified to "haul out a tooth."

Once when I was upon the sea shore I was called upon to "haul out a tooth" for a sturdy sailor. I "hauled" at it, but it would not "haul." I "hauled again." Again it would not—a third effort and I "hauled it out." He said I could beat any man "a hauling on 'em" he ever saw; I

felt complimented, thought I had not mistaken my "*forte*," and that my worthy preceptor had shown great sagacity *on the selection of his students!* I thank you gentlemen for your kind attention.

